# Metabolite and Transcriptome Profiling Analysis Revealed That Melatonin Positively Regulates Floral Scent Production in *Hedychium coronarium*

**DOI:** 10.3389/fpls.2021.808899

**Published:** 2021-12-17

**Authors:** Farhat Abbas, Yiwei Zhou, Jingjuan He, Yanguo Ke, Wang Qin, Rangcai Yu, Yanping Fan

**Affiliations:** ^1^Research Center for Ornamental Plants, College of Forestry and Landscape Architecture, South China Agricultural University, Guangzhou, China; ^2^College of Economics and Management, Kunming University, Kunming, China; ^3^College of Life Sciences, South China Agricultural University, Guangzhou, China; ^4^Guangdong Key Laboratory for Innovative Development and Utilization of Forest Plant Germplasm, South China Agricultural University, Guangzhou, China

**Keywords:** *Hedychium coronarium*, metabolome, transcriptome, melatonin, floral scent

## Abstract

Melatonin is a pleiotropic molecule that regulates a variety of developmental processes. Floral volatiles are important features of flowers that facilitate flower–visitor interactions by attracting pollinators, structure flower–visitor communities, and play defensive roles against plant and flower antagonists. Aside from their role in plants, floral volatiles are an essential ingredient in cosmetics, perfumes, pharmaceuticals, and flavorings. Herein, integrated metabolomic and transcriptomic approaches were carried out to analyze the changes triggered by melatonin exposure during the *Hedychium coronarium* flower development stages. Quantitative analysis of the volatiles of *H*. *coronarium* flowers revealed that volatile organic compound emission was significantly enhanced after melatonin exposure during the half bloom (HS), full bloom (FB) and fade stage (FS). Under the melatonin treatment, the emission of volatile contents was highest during the full bloom stage of the flower. Variable importance in projection (VIP) analysis and partial least-squares discriminant analysis (PLS-DA) identified 15 volatile compounds with VIP > 1 that were prominently altered by the melatonin treatments. According to the transcriptome sequencing data of the HS, FB, and FS of the flowers, 1,372, 1,510, and 1,488 differentially expressed genes were identified between CK-HS and 100MT-HS, CK-FB and 100MT-FB, and CK-FS and 100MT-FS, respectively. Among the significant differentially expressed genes (DEGs), 76 were significantly upregulated and directly involved in the floral scent biosynthesis process. In addition, certain volatile organic compounds were substantially linked with various DEGs after combining the metabolome and transcriptome datasets. Moreover, some transcription factors, such as MYB and bHLH, were also significantly upregulated in the comparison, which might be related to the floral aroma mechanism. Our results suggested that melatonin increased floral aroma production in *H*. *coronarium* flowers by modifying the expression level of genes involved in the floral scent biosynthesis pathway. These findings serve as a foundation for future research into the molecular mechanisms underlying the dynamic changes in volatile contents induced by melatonin treatment in *H*. *coronarium*.

## Introduction

Melatonin (*N*-acetyl-5-methoxytryptamine) is a low molecular weight molecule that is ubiquitously present in nature, and it has pleiotropic cellular and physiological activities in several kingdoms (Tan et al., 2012; Zhao et al., 2019). It was first discovered in the cow pineal gland in 1958, where it acts as a neurohormone and contributes to the regulation of numerous physiological processes, including circadian rhythm, appetite, body temperature, immunological system, etc (Lerner et al., 1958; Jan et al., 2009; Carrillo-Vico et al., 2013). Melatonin is also engaged in a variety of cellular functions as an antioxidant, and it has good *in vitro* and *in vivo* free radical scavenging activities (Fischer et al., 2013; Arnao and Hernández-Ruiz, 2015). Furthermore, it is considered a master regulator of plant growth, gene expression developmental regulation and stress alleviation. Melatonin plays a significant role in the metabolism of ethylene, indole-3-acetic acid, gibberellin, cytokinin, and auxin carrier proteins, as well as other plant hormones (Li et al., 2019; Nawaz et al., 2021). It also extends the shelf life and quality of fruits and vegetables and plays a key role as an anti-sensor agent in response to numerous abiotic stresses, such as salinity, temperature, drought, UV radiation, and harmful substances (Arnao and Hernández-Ruiz, 2006, 2019). Melatonin’s function in plants has been intensively explored in recent years, although its role in the regulatory mechanism of floral aroma synthesis is completely unknown.

Floral volatiles have piqued human interest, and this appeal has spread to various parts of human life. Floral scent is widely used for perfumes, cosmetics, flavorings, and therapeutic applications. Recently, several compounds of terpenes, benzenoids/phenylpropanoids, derivatives of fatty acids are been extensively used in aforementioned cosmetics and pharmaceuticals industry (Dudareva et al., 2006). Moreover, floral volatiles play crucial roles in maintaining the ecological linkage between flowers and a diverse range of visitors, such as pollinators, florivores and pathogens, hence playing a key role in plant reproduction and evolutionary variety (Dudareva et al., 2013; Nagegowda and Gupta, 2020). The complex biochemical pathway underlying the synthesis of floral aroma is led by various internal and external stimuli, thus allowing the controlled emission of volatiles (Aharoni et al., 2005; Dudareva and Pichersky, 2008; Abbas et al., 2017). The presence of volatiles in ornamental plants plays a decisive role in pollinator attraction and customers’ esthetic preferences (Raguso, 2009). The quality of flower scent is determined by complex lipophilic molecules with low molecular weight that are generated by terpenoid, phenylpropanoid/benzenoid, and fatty acid biosynthetic pathways (Pichersky et al., 2006; Muhlemann et al., 2014).

White garland or ginger lily (*Hedychium coronarium*) is a perennial flowering plant that belongs to the family Zingiberaceae. It is an economically significant crop that has been extensively cultivated for ornamental, medicinal and aromatic oil production purposes. *H. coronarium* has long strap-like leaves and large spikes of fragrant white flowers that can grow up to 25 cm long. *H*. *coronarium* flowers release a huge number of volatile chemicals, including monoterpenes, sesquiterpenes and benzenoids (Fan et al., 2003; Ke et al., 2019; Abbas et al., 2021a,b; Zhou et al., 2021). Several structural genes encoding terpene synthases and benzoic/salicylic acid methyltransferase have previously been characterized in the *H*. *coronarium* volatile biosynthesis pathway. However, less is known about the transcriptional regulation of floral aroma synthesis and emission.

The gas chromatography–mass spectrometry (GC–MS) technique has been widely used in metabolic profiling studies of plants. Recently, the GC–MS approach has been used to identify and quantify the volatile profiles of numerous flowering plants, including *Lilium* ‘Siberia’ (Abbas et al., 2019, 2020a,b, 2021c,d), *H*. *coronarium* (Fan et al., 2007; Zhou et al., 2021), *Silene latifolia* (Dötterl et al., 2005), *Rosa damascene* (Rusanov et al., 2011), *Osmanthus fragrans* (Cai et al., 2014), *Panax ginseng* (Lee et al., 2017), *Lantana canescens* Kunth (Pino et al., 2011), and *Luculia pinceana* (Li et al., 2016). However, integrative transcriptome and metabolome analyses have been used as a unique tool for elucidating the mechanisms that regulate metabolic networks in plants (Fukushima et al., 2014). First, high-throughput sequencing and non-targeted metabolome analysis were used to examine genes that regulate metabolic pathways and related chemicals. The findings were then analyzed to elucidate gene metabolite correlations and identify genes with unknown functions. This technique has been used to investigate the coordinated regulation of genes and metabolites involved in phenylpropanoid metabolism in *Arabidopsis thaliana* (Tohge et al., 2005), *Populus* × *canescens* (Behnke et al., 2010) and *Medicago truncatula* (Farag et al., 2008). Furthermore, comparative transcriptome and metabolome analyses have been performed in *M*. *truncatula* (Suzuki et al., 2002), *Papaver somniferum* (Zulak et al., 2007), and *Catharanthus roseus* (Rischer et al., 2006) to study alkaloid and triterpene metabolism.

In the current study, integrated metabolomes and transcriptomes obtained through headspace solid phase microextraction (HS–SPME) GC–MS and RNA sequencing analyses of flowers after exogenous melatonin application were employed. To gain a comprehensive picture of the main metabolic pathways and associated genes, we compared data from the same tissues using various bioinformatics analyses. We first revealed putative regulatory role of melatonin in floral aroma enhancement. This study will shed light on the novel regulatory function of melatonin in floral aroma synthesis and the gene regulation network of floral volatile biosynthesis pathways in *H*. *coronarium*.

## Materials and Methods

### Plant Materials and Growth Conditions

*Hedychium coronarium* was grown in a growth chamber under the following conditions: 24 ± 2°C, 70–80% and a 12 h/12 h day/night photoperiod. For tissue-specific expression patterns, plant parts, including flowers, leaves and rhizomes, were isolated, immediately frozen in liquid nitrogen and stored at −80°C. The flower development process was separated into three stages: bud stage, full bloom, and senescence.

### Melatonin Treatments

The stems of the flowers were cut into 40 cm lengths and immersed in sterile water containing 50, 100, 500, and 1,000 μM melatonin (MT) for the hormone treatment. The melatonin stock solution was prepared following the instructions suggested by the manufacturers. Briefly, melatonin (0.2323 g) powder was dissolved in 2 mL methanol and diluted in sterilized water at the abovementioned concentrations. Detached flowers were then placed in different glass beakers that contained different melatonin solutions (50, 100, 500, and 1,000 μM). The control flowers were placed in an equal volume of sterilized water without melatonin under the same conditions as described above. After analyzing the volatile contents, the flower samples were frozen in liquid nitrogen and stored at -80°C for RNA sequence analysis. Four to five independent trials were performed with each experimental variant.

### Headspace Volatile Collection and Gas Chromatography–Mass Spectrometry Analysis

The collection of headspace floral volatiles and GC–MS analysis were carried out as previously described (Yue et al., 2014; Ke et al., 2021). In short, entire flowers from each stage were placed in a 500 mL glass bottle, and 1.728 μg (microgram) ethyl caprate was added as an internal standard. The glass bottle was stilled for 30 min, and then a polydimethylsiloxane (50/30 μm divinylbenzene/carboxen) fiber (Supelco) was injected into the glass bottle to trap volatiles for 30 min. Thereafter, the adsorbed volatile compounds were analyzed using a GC–MS system with an Agilent 7890A GC and Agilent 5975C MSD as previously explained (Yue et al., 2015, 2021).

### Gas Chromatography–Mass Spectrometry Conditions and Identification of Floral Volatiles

The GC–MS system was furnished with an Agilent DB-5MS capillary column (30 m × 0.25 mm), and helium gas was used as a carrier at a persistent flow of 1 mL/min. First, the oven temperature was kept at 40°C for 2 min, increased to 250°C at a rate of 5°C/min, and held for 5 min at 250°C. The identification of floral volatiles was performed by comparing the retention time and mass spectra with authentic standards. The quantification of floral volatiles was measured using the Agilent ChemStation Data Analysis Application based on the peak areas and quantity of internal standard. The floral VOCs were identified by comparing them with mass spectra from the NIST Mass Spectral Library (NIST 08). The identification of compounds was perceived by comparing the mass spectra with NIST 08 at a match factor of ≥80. Linear retention indices (LRIs) of the volatile compounds were measured via an alkane series standard (C7–C40) (Sigma, St. Louis, MO, United States).

### RNA-Seq

Total RNA extraction and other experimental details were performed by Novogene (UK Sequencing Center, Cambridge, United Kingdom). The experimental details were derived from Novogene’s data. Quality control and RNA-seq were performed by Novogene, and high-quality samples were ensured. The library was built from total RNA, and RNA sequencing was performed by Illumina (San Diego, CA, United States) platforms using the SBS mechanism (sequencing by synthesis). Then, a bioinformatics analysis was carried out. The treatments were analyzed and compared to each other. The detailed methodology provided by Novogene is included in the Supplementary Material. For RNA sequencing, the cDNA library was generated using an Illumina HiSeq™ RNA Sample Prep Kit (Illumina, United States) following the manufacturer’s protocols. The size and concentration of the library were evaluated using Qubit 2.0 and Agilent 2100. Through the HiSeq 2500 sequencing machine, paired-end (150 PE) Illumina high-throughput sequencing was performed.

### Gene Functional Annotation and Expression Level Analysis

From the raw reads, the low-quality reads and adopters were filtered. The clean reads were de novo assembled into contigs with an optimized k-mer length = 25 and group pair distance = 300 using the Trinity program 2. The unigene functions were predicted via BLAST against the NCBI non-redundant protein (Nr), NCBI nucleotide sequences (Nt), and Swiss-Prot databases (*E*-value of 10^–5^). The resulting datasets were validated to the Protein family (Pfam) database (Finn et al., 2016) with HMMER (*E*-value 10^–10^). Unigene sequences were aligned against the Gene Ontology (GO) (Ashburner et al., 2000), Kyoto Encyclopedia of Genes and Genomes (KEGG) (Kanehisa et al., 2004), and euKaryotic Orthologous Groups (Koonin et al., 2004) databases. The expression levels of genes in the samples were measured via the FPKM procedure. Based on gene read count data, DEGseq software was employed to identify differentially expressed genes (DEGs). The following criteria were established for screening DEGs: | log2 (fold change)| ≥ 1 and false discovery rate (FDR) < 0.05.

### Quantitative Real-Time PCR and Expression Validation

To validate the RNA-seq data, quantitative real-time PCR (qRT–PCR) was performed as described previously (Abbas et al., 2019, 2020a,b). Isolation of total RNA was performed using a HiPure Plant RNA Mini Kit (Magen) following the manufacturer’s guidelines. The concentration of RNA was calculated with a spectrophotometer. Conversion of total RNA into cDNA was carried out using the PrimeScript RT Reagent Kit (TaKaRa) according to the manufacturer’s suggestions. Approximately 1 μg of total RNA was reverse transcribed by a PrimeScriptTM RT reagent kit according to the manufacturer’s protocol. qRT–PCR analysis was performed using the ABI 7500 Fast Real-Time PCR System (Applied Biosystems, United States) with a 96-well plate. The reaction volume (20 μL) contained 10 μL pink SYBR Supermix, 0.4 μL of each forward and reverse primer, 7.2 μL of ddH_2_O and 2 μL of cDNA. GAPDH was selected as an internal reference control, and the assay was performed in three technical and three biological replicates. The relative expression level of genes was measured according to the 2^–ΔΔCT^ method (Livak and Schmittgen, 2001).

### Data Analysis

SPSS 19.0 (SPSS Inc., Chicago, IL, United States) was used for the statistical analyses and analysis of variance (ANOVA). A correlation analysis was based on the Spearman correlation coefficient using R 4.1.1. The PLS-DA was implemented with the “mixOmics” and “RVAideMemoire” packages of R (4.1.1). The regression coefficient network was constructed using Cytoscape version 3.6.2 (Cline et al., 2007). Identification of transpose elements (TE) was performed using the Protein-based RepeatMasking^1^ with a *e*-value of 0.00001. The mean FPKM of different TE were calculated from the transcriptome data.

## Results

### Effect of Melatonin on Floral Scent Emission in *H*. *coronarium* Flowers

**FIGURE 1:**
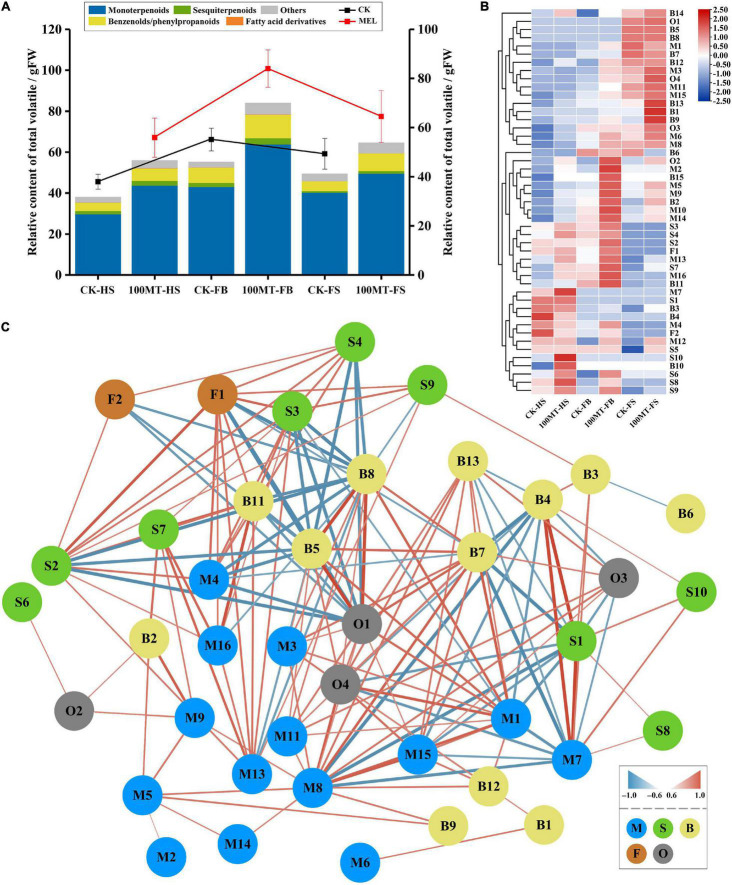
Content and type of volatile compounds identified by HS–SPME–GC–MS in *H*. *coronarium*. **(A)** Changes in the total floral volatile contents of *H*. *coronarium* flowers between melatonin-treated and control flowers. **(B)** Heatmap of volatile compounds identified between control and treated flowers. **(C)** Spearman correlation of identified volatile compounds detected by HS–SPME–GC–MS based on significance (*p* < 0.05). The gradient color coding of the edges and the line thickness denote the level of correlation (0.6–1). Data are shown as the ± SEM value of five repeats.

To evaluate the differences in volatile organic compounds (VOCs) between treated and untreated flowers during different developmental stages, an HS–SPME–GC–MS approach was employed. *Hedychium coronarium* flowers were treated with 100 μM melatonin solution. The data showed that compared to the control flowers (non-treated flowers), melatonin-treated flowers exhibited a significantly high level of floral scent emission (Figure 1). Under the melatonin treatment, the emission of floral volatile contents was increased by 47.3% during the half bloom stage of the flower compared to flowers not treated with melatonin. Similarly, the emission level of volatile contents was increased by 52.36 and 30.86% during the full bloom stage and senescence stage, respectively, compared to the control flowers.

Volatile organic compounds and their relative quantities emitted during flower development are summarized in Table 1. During the flower developmental process, 47 VOCs were identified, including 16 monoterpenes, 10 sesquiterpenes, 15 benzenoids/phenylpropanoids, 2 fatty acid derivatives, and 4 other categories (Table 1 and Figure 1A). The relative quantities and kinds of VOCs in the flowers showed substantial differences. Monoterpenes and sesquiterpenes were the main floral volatile compounds of *H. coronarium*, which is in line with our previous findings. The total volatile contents of monoterpenes were increased by 46.91, 48.3, and 22.94% during the half bloom, full bloom and senescence stages, respectively, compared to the control. During the half bloom, full bloom and senescence stages, the total volatile contents of sesquiterpenes increased by 41.61, 49.51, and 63.64%, respectively. Similarly, the total volatile contents of benzenoids/phenylpropanoids increased up to 53.75, 53.97, and 83.97% during the half bloom, full bloom and senescence stages, respectively. Interestingly, the contents of fatty acid derivates increased by 100% at the full bloom stage of the flowers after melatonin treatment but did not change during the bud and senescence stages.

**TABLE 1:** Changes in floral volatile profiles of *H. coronarium* flowers during different flower development processes under melatonin application.

Compound name	Code	Chemical formula	RI1	RI2	MS	CK-HB	100MT-HB	CK-FB	100MT-FB	CK-FS	100MT-FS
2-methylpropionamide oxime	O1	C4H9NO	742	–	90	–	–	–	–	0.16 ± 0.03^a^	0.18 ± 0.05 a
Butanal, 3- methyl-, oxime	O2	C5H11NO	849	–	87	2.24 ± 0.46 c	3.41 ± 0.75 b	2.21 ± 0.33 c	4.88 ± 0.86 a	2.65 ± 0.65 c	3.79 ± 0.25 b
α-thujene	M1	C10H16	925	923	90	0.06 ± 0.02 c	0.06 ± 0.02 c	0.08 ± 0.02 bc	0.12 ± 0.01 b	0.18 ± 0.05 a	0.15 ± 0.03 ab
α-pinene	M2	C10H16	932	937	95	0.17 ± 0.02 b	0.23 ± 0.1 b	0.2 ± 0.04 b	0.33 ± 0.06 a	0.22 ± 0.03 b	0.22 ± 0.04 b
β-thujene	M3	C10H16	972	966	91	0.23 ± 0.1 b	0.2 ± 0.04 b	0.23 ± 0.12 b	0.4 ± 0.15 a	0.44 ± 0.1 a	0.54 ± 0.16 a
β-pinene	M4	C10H16	977	979	95	0.68 ± 0.3 a	0.55 ± 0.39 a	0.38 ± 0.07 b	0.66 ± 0.15 a	0.17 ± 0.05 b	0.2 ± 0.07 b
β-myrcene	M5	C10H16	989	990	91	0.76 ± 0.22 a	1 ± 0.18 a	1.06 ± 0.16 a	1.67 ± 0.06 a	1.11 ± 0.28 a	1.39 ± 0.16 a
α-terpinene	M6	C10H16	1017	1017	96	0.05 ± 0.02 c	0.07 ± 0.01 bc	0.08 ± 0.02 bc	0.1 ± 0.03 ab	0.09 ± 0.05 bc	0.11 ± 0.02 a
O-cymene	B1	C10H14	1026	1030	83	0.02 ± 0.01 a	0.03 ± 0 a	0.03 ± 0.01 a	0.05 ± 0 a	0.05 ± 0.04 a	0.2 ± 0.16 a
D-limonene	M7	C10H16	1029	1029	94	0.18 ± 0.04 b	0.4 ± 0.38 a	–	–	–	–
Eucalyptol	M8	C10H18O	1032	1033	97	1.25 ± 0.36 d	3.2 ± 1.12 c	6.31 ± 1.09 b	10.2 ± 1.25 a	10.14 ± 0.68 a	10.97 ± 2.11 a
(Z)-β-ocimene	M9	C10H16	1048	1040	97	13.2 ± 1.98 c	19.39 ± 4.57 b	19.94 ± 2.58 b	28.8 ± 1.67 a	19.59 ± 5.21 b	22.59 ± 4.28 b
Cyclopentene, 3-isopropenyl-5,5-dimethyl-	M10	C10H16	1057	–	95	0.05 ± 0.02 b	0.06 ± 0.03 ab	0.07 ± 0.03 ab	0.09 ± 0.02 a	0.06 ± 0.03 ab	0.07 ± 0.01 ab
γ-terpinene	M11	C10H16	1059	1058	96	0.05 ± 0.02 c	0.05 ± 0.01 c	0.06 ± 0.03 c	0.07 ± 0.01 bc	0.09 ± 0.03 ab	0.1 ± 0.02 a
Terpinolene	M12	C10H16	1086	1085	96	0.1 ± 0.01 a	0.1 ± 0.02 a	0.09 ± 0.04 a	0.1 ± 0.03 a	0.09 ± 0.03 a	0.1 ± 0.02 a
Methyl benzoate	B2	C8H8O2	1094	1095	95	3.2 ± 0.71 e	5.13 ± 1.36 cd	6.05 ± 1.41 bc	9.42 ± 1.13 a	3.92 ± 0.8 de	7.52 ± 1.9 b
Linalool	M13	C10H18O	1099	1102	97	12.45 ± 2.88 c	17.72 ± 1.4 ab	13.77 ± 2.72 bc	20.35 ± 4.28 a	7.42 ± 1.91 d	12.32 ± 2.1 c
(E,E)-cosmene	O3	C10H14	1124	1130	96	0.02 ± 0.01 c	0.03 ± 0 bc	0.04 ± 0.01 ab	0.04 ± 0 ab	0.04 ± 0.01 ab	0.05 ± 0.01 a
Allo-ocimene	M14	C10H14	1131	1131	97	0.5 ± 0.11 c	0.64 ± 0.08 bc	0.73 ± 0.1 ab	0.89 ± 0.11 a	0.65 ± 0.17 bc	0.73 ± 0.15 ab
Benzyl nitrile	B3	C8H7N	1140	1150	96	0.18 ± 0.07 a	0.17 ± 0.02 ab	0.11 ± 0.02 c	0.12 ± 0.02 bc	0.08 ± 0.05 c	0.13 ± 0.03 bc
Methyl salicylate	B4	C8H8O3	1193	1191	91	0.09 ± 0.12 a	0.04 ± 0.02 a	–	–	–	–
α-terpineol	M15	C10H18O	1196	1199	87	0.02 ± 0.01 c	0.03 ± 0.01 bc	0.04 ± 0.01 bc	0.05 ± 0.01 ab	0.06 ± 0.01 ab	0.07 ± 0.03 a
Geraniol	M16	C10H18O	1251	1256	86	0.01 ± 0.01 bc	0.02 ± 0 ab	0.02 ± 0.01 a	0.03 ± 0.01 a	0.01 ± 0 c	0.01 ± 0.01 bc
Propanoic acid, phenylmethyl ester	B5	C10H12O2	1258	–	91	–	–	–	–	0.01 ± 0 a	0.01 ± 0 a
Indole	O4	C8H7N	1292	1290	97	0.17 ± 0.14 d	0.16 ± 0.12 d	0.27 ± 0.03 cd	0.56 ± 0.26 bc	0.63 ± 0.2 ab	0.96 ± 0.5 a
Benzoic acid, 2- methoxy-, methyl ester	B6	C9H10O3	1333	1336	83	0.01 ± 0 b	0.01 ± 0 b	0.07 ± 0.02 a	0.06 ± 0.06 a	0.07 ± 0.05 a	0.02 ± 0.01 b
δ-elemene	S1	C15H24	1340	1339	90	0.01 ± 0 a	0.01 ± 0 a	–	–	–	–
Methyl anthranilate	B7	C8H9NO2	1344	1346	96	–	–	0.02 ± 0.01 b	0.02 ± 0.01 b	0.07 ± 0.05 a	0.06 ± 0.04 a
Butanoic acid, phenylmethyl ester	B8	C11H14O2	1347	1349	94	–	–	–	–	0.01 ± 0 a	0.01 ± 0 a
Eugenol	B9	C10H12O2	1354	1356	98	0.02 ± 0.01 b	0.03 ± 0.01 b	0.03 ± 0.01 b	0.04 ± 0.01 ab	0.03 ± 0.02 b	0.07 ± 0.04 a
Methyl cinnamate	B10	C10H10O2	1387	1397	95	–	0.02 ± 0.01 a	0.01 ± 0 c	0.01 ± 0 ab	0.01 ± 0 ab	0.01 ± 0 b
Caryophyllene	S2	C15H24	1429	1420	99	0.38 ± 0.13 b	0.36 ± 0.11 b	0.34 ± 0.1 b	0.6 ± 0.24 a	0.05 ± 0.05 c	0.05 ± 0.01 c
1-Butanol, 3- methyl-, benzoate	B11	C12H16O2	1442	1441	83	0.32 ± 0.23 c	0.42 ± 0.08 c	0.91 ± 0.29 b	1.35 ± 0.65 a	0.06 ± 0.02 c	0.2 ± 0.09 c
Trans-isoeugenol	B12	C10H12O2	1450	1448	98	0.08 ± 0.05 b	0.17 ± 0.11 ab	0.1 ± 0.01 b	0.23 ± 0.14 ab	0.28 ± 0.1 a	0.28 ± 0.19 a
(E)-β-famesene	S3	C15H24	1454	1456	94	0.3 ± 0.18 b	0.41 ± 0.18 ab	0.34 ± 0.15 ab	0.52 ± 0.28 a	0.04 ± 0.04 c	0.04 ± 0.01 c
Humulene	S4	C15H24	1466	1453	97	0.04 ± 0.01 bc	0.06 ± 0.03 ab	0.05 ± 0.01 ab	0.06 ± 0.02 a	0.02 ± 0.01 cd	0.02 ± 0.01 d
Alloaromadendrene	S5	C15H24	1470	1461	99	0.02 ± 0.01 a	0.02 ± 0.01 a	0.02 ± 0 a	0.02 ± 0.01 a	0.01 ± 0.01 a	0.02 ± 0.01 a
Germacrene d	S6	C15H24	1482	1477	91	0.01 ± 0 ab	0.02 ± 0.01 a	–	0.02 ± 0 a	0.01 ± 0 b	0.01 ± 0.01 a
Jasmine lactone	F1	C10H16O2	1492	–	96	0.1 ± 0.04 ab	0.12 ± 0.04 a	0.07 ± 0.04 b	0.15 ± 0.04 a	–	–
Benzene, 1,2-dimethoxy-4-(1-propenyl)-	B13	C11H14O2	1494	1492	96	0.02 ± 0.01 b	0.04 ± 0.02 b	0.05 ± 0.03 b	0.05 ± 0.01 b	0.06 ± 0.01 b	0.11 ± 0.07 a
Benzyl tiglate	B14	C12H14O2	1500	1498	94	0.05 ± 0.03 ab	0.07 ± 0.04 ab	0.03 ± 0.01 b	0.06 ± 0.01 ab	0.07 ± 0.02 a	0.08 ± 0.03 a
α-farnesene	S7	C15H24	1505	1524	97	0.78 ± 0.17 cd	1.29 ± 0.23 b	1.24 ± 0.25 b	1.75 ± 0.21 a	0.6 ± 0.31 d	1.07 ± 0.28 bc
α-amorphene	S8	C15H24	1521	1519	97	0.03 ± 0.01 ab	0.04 ± 0.02 a	0.02 ± 0.01 b	0.03 ± 0.01 b	0.02 ± 0.01 b	0.02 ± 0.01 b
δ-cadinene	S9	C15H24	1524	1525	96	0.04 ± 0.01 ab	0.05 ± 0.02 a	0.03 ± 0.01 b	0.05 ± 0.02 ab	0.02 ± 0.01 c	0.03 ± 0.01 bc
Nerolidol	S10	C15H26O	1563	1562	90	–	0.02 ± 0.01	–	–	–	–
Methyl jasmonate	F2	C13H20O3	1681	1638	96	0.05 ± 0.03 a	0.03 ± 0 ab	0.02 ± 0.01 bc	0.03 ± 0.01 ab	0.01 ± 0 c	0.01 ± 0 c
Benzyl benzoate	B15	C14H12O2	1780	1760	96	0.01 ± 0.01 a	0.02 ± 0.01 a	0.02 ± 0 a	0.03 ± 0.01 a	0.02 ± 0.01 a	0.02 ± 0.02 a

*^1^RT, real time.*

*^2^LRI calc, the calculated linear retention indices.*

*^3^LRI Nist, linear retention indices in the literature; Column phase type, DB-5MS.*

*^4^MS, mass spectrum comparison using NIST libraries. Figures in the table are means and standard error.*

*a, b, c, d, means within a row refer to the significant difference (p < 0.05).*

*Different letters indicate significant differences among means according to ANOVA analysis (p < 0.05).*

Moreover, some VOCs were development-specific and released during the specific flower development stage (Figure 1B). For example, the emission of D-limonene, methyl salicylate and δ-elemene was only observed during the half bloom stage of the flower, while 2-methylpropionamide oxime, propanoic acid, phenylmethyl ester and butanoic acid, phenylmethyl ester were only emitted during the senescence stage of the flower. Similarly, methyl anthranilate was released during the full bloom and senescence stage of the *H. coronarium* flowers. Furthermore, a correlation network analysis of total and major differentially accumulated metabolites (DAMs) that produced floral volatile content between the treated and untreated flowers was performed (Supplementary Figure 1 and Figure 1C). The analysis showed that among differentially accumulated metabolites (DAMs), monoterpenes were the foremost compounds interlinked with other floral volatiles (Figure 1C). Among DAMs, 2-methylpropionamide oxime was connected with the maximum number (17) of nodes, followed by α-thujene (14), eucalyptol (12), D-limonene (11), linalool (9), propanoic acid, phenylmethyl ester (9), and β-pinene (8). Overall, the results showed that several compounds presented a significant response to melatonin treatments.

### Identification of the Important Volatile Organic Compounds Influenced by Melatonin Treatment

**FIGURE 2:**
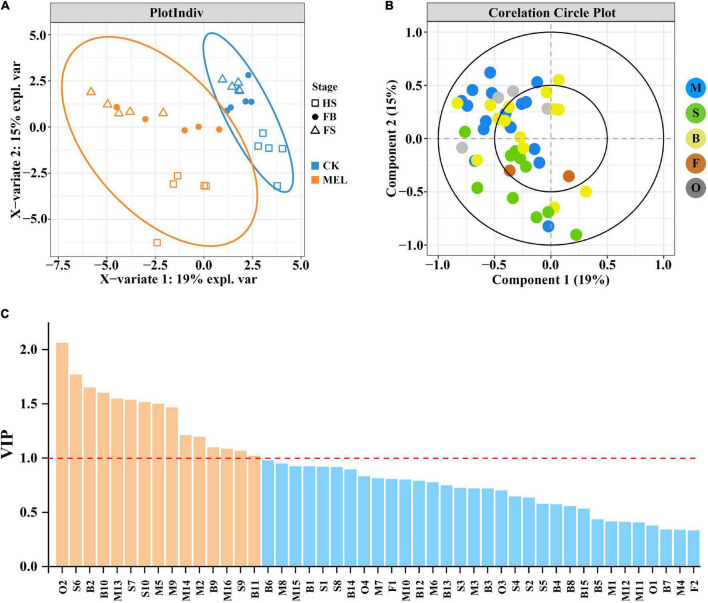
PLS-DA correlation analysis of volatile compounds detected by HS–SPME–GC–MS. **(A)** Plot indicating the correlation analysis of melatonin-treated samples and control samples during three flower development stages. **(B)** Plot showing the loading plot of the PLS-DA analysis of volatile compounds measured by HS–SPME–GC–MS. **(C)** Variables capable of discriminating volatile compounds identified between melatonin-treated and control flowers are shown, as ordered by VIP score. VIP scores ≥1 (above the red line) identified key variables for predicting Y responses (relapse).

Partial least squares-discriminant analysis (PLS-DA) is an effective and highly reliable approach for the discriminative variable selection and descriptive and predictive modeling. This technique is useful to clarify the distinction between groups of observations and aids in elucidating which variables transmit class-defining information. The PLS-DA results showed that metabolite profiles in three flower development stages were substantially modified (Figure 2). As shown in Figure 2A, there was a significant difference between the samples before and after melatonin treatment. The PLS-DA score plot model discriminated the flower development stages, with five samples exhibiting satisfactory distance. Furthermore, this finding reflects that all stages have substantially different metabolite compositions. Figure 2B shows that component 2 was the most important variable and positively correlated with most of the monoterpenes and benzenoids/phenylpropanoid compounds. These results showed that the production of most volatile compounds was positively correlated with the melatonin treatment.

Figure 2C shows the data for variable importance in projection (VIP), which is a weighted sum of squares of the PLS-DA loadings based on the amount of explained Y variation in each dimension. The results showed that 15 volatile compounds, including 6 monoterpenes (linalool, β-myrcene, (Z)-β-ocimene, allo-ocimene, α-pinene and geraniol), 4 sesquiterpenes (germacrene D, α-farnesene, nerolidol, and δ-cadinene), 4 benzenoids/phenylpropanoids (benzoic acid, methyl cinnamate, eugenol and 1-butanol, 3- methyl-, and benzoate) and one other compound (butanal, 3- methyl-, and oxime) with VIP > 1, were identified by PLS-DA and were substantially influenced by the melatonin treatments. Briefly, the results showed that these 15 compounds were greatly influenced by the melatonin treatment and played key roles during floral aroma production in *H*. *coronarium*.

### Transcriptome Analysis of *H*. *coronarium* Flowers Under the Melatonin Treatment

We performed a transcriptome analysis on *H*. *coronarium* treated with exogenous melatonin to investigate the molecular mechanism of exogenous melatonin in ginger plants. Information regarding the sequencing and assembly is provided in Supplementary Table 1. RNA-seq data (PRJNA777930) resulted in 6.04–7.52 GB clean bases with an error ratio of less than 0.02%, a Q20 value higher than 98.2%, a Q30 value greater than 94.44%, and a GC percentage ranging from 46.04 to 50.13% (Supplementary Table 1). The total mapped reads ranged from 94.14 to 95.67%, which included more than 83.68% unique mapped reads (Supplementary Table 2). Briefly, the data suggested that sequences were of high quality and met the requirements for further analysis.

### Analysis of Differentially Expressed Genes in *H*. *coronarium* Flowers Under the Melatonin Treatment

**FIGURE 3:**
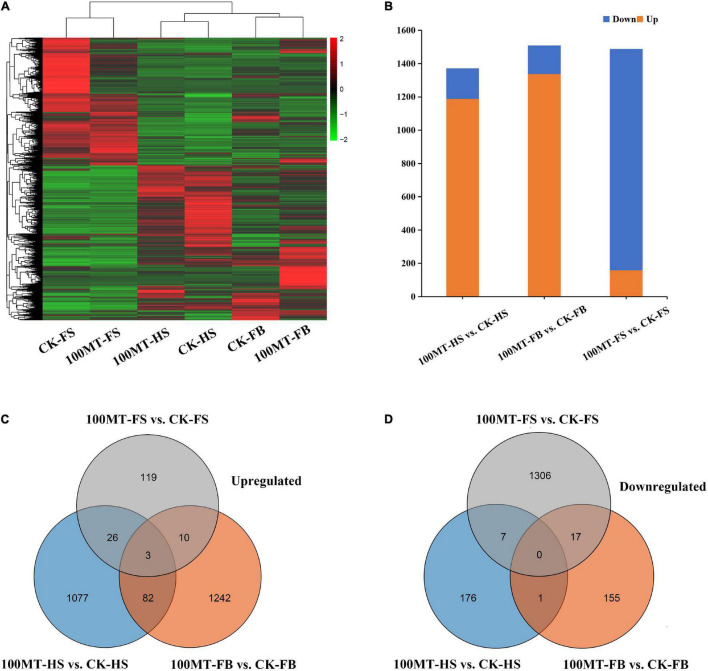
Melatonin’s effects on differentially expressed genes (DEGs). **(A)** Heatmap representation of DEGs tempted by melatonin. **(B)** Number of upregulated and downregulated genes among DEGs. Venn diagram of the upregulated **(C)** and downregulated **(D)** DEGs.

In this study, a total of 37,937 differentially expressed genes were found, and the length of the genes varied from 81 bp to 20,934 bp (Figure 3A). The DEGs for different comparisons were identified by DESeq based on the FPKM (fragments per kilobase per million mapped reads) values, and DEGs with log2 (fold change) ≥ 1 and FDR < 0.05 were considered significantly different. A total of 1,188, 1,337, 158, 538, 4,451, and 3,921 genes were upregulated and 184, 173, 1,330, 1,623, 3,917, and 2,200 genes were downregulated in the half stage ck (CK-HS) vs. half stage treatment (100MT-HS), full bloom ck (CK-FB) vs. full bloom treatment (100MT-FB), fade stage ck (CK-FS) vs. fade stage treatment (100MT-FS), 100MT-HS vs. 100MT-FB, 100MT-HS vs. 100MT-FS, and 100MT-FB vs. 100MT-FS comparisons, respectively (Figure 3B). Overall, all six abovementioned comparisons had 1,372, 1,510, 1,488, 2,161, 8,368, and 6,121 DEGs, respectively. In short, these results indicate that there was a significant difference in the gene expression levels after treatment.

A Venn diagram analysis showed that in the CK-HS vs. 100MT-HS and CK-FB vs. 100MT-FB comparisons, the number of upregulated and downregulated genes was significantly higher than the number of downregulated genes, while the opposite trend was observed in CK-FS vs. 100MT-FS. The number of downregulated genes was higher than that of upregulated genes (Supplementary Figure 2). The number of upregulated and downregulated DEGs in each comparison and their overlapping relationship were evaluated (Figures 3B–D). Overall, 1,376 genes (1,188 genes were upregulated and 184 were downregulated compared to CK-HS) were significantly changed between CK-HS and 100MT-HS. A total of 1,510 genes (1,337 genes were upregulated and 173 were downregulated compared to CK-FB) were differentially expressed between CK-FB and 100MT-FB. Similarly, 1,488 genes (158 genes were upregulated and 1,330 were downregulated compared to CK-FB) were substantially modified between CK-FS and 100MT-FS. Overall, the number of DEGs overlapping in the CK-HS vs. 100MT-HS, CK-FB vs. 100MT-FB, and CK-FS vs. 100MT-FS comparisons was 119, 113, and 63, respectively. Furthermore, transpose element analysis was performed to identify the significantly influenced transpose super-families under melatonin treatment compared to control. Results showed that 57 type of transpose elements were identified from the transcriptome data which showed significant up and downregulated expression under MT treatment (Supplementary Figure 3). Among different transpose super-families, DNA, LINE and LTR super-families were abundant and significantly influenced by MT treatment.

### Gene Ontology and Kyoto Encyclopedia of Genes and Genomes Annotation and Enrichment Analysis of Differentially Expressed Genes

**FIGURE 4:**
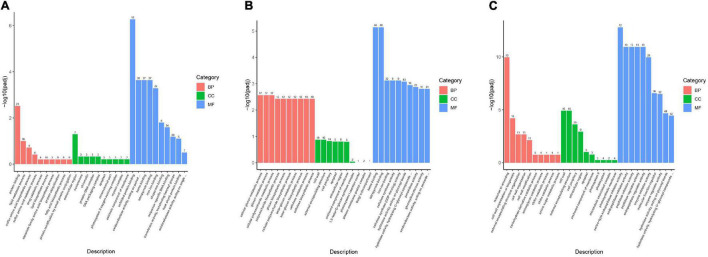
GO classification among the **(A)** CK-HS vs. 100MT-HS, **(B)** CK-FB vs. 100MT-FB and **(C)** CK-FS vs. 100MT-FS comparisons. Biological processes (BP), cellular component (CC), and molecular function (MF) are indicated in red, green, and blue colors. The number of DEGs are shown on the bar.

The function of the DEGs was investigated using GO-based enrichment annotation, which was classified into three key functional categories: biological process (BP), molecular function (MF), and cellular component (CC) (Figures 4A–C). Between CK-HS and 100MT-HS, 2,393 DEGs had GO annotations, most of which were classified as protein folding (GO: 0006457), extracellular region (GO: 0005576) and unfolded protein binding (GO: 0051082) (Figure 4A). All CK-FB vs. 100-FB DEGs were annotated to the GO database, and the majority were highly enriched in “cellular glucan metabolic process (GO: 0006073), cell wall (GO: 0005618) and heme binding (GO: 0020037) (Figure 4B and Supplementary Table 3). Similarly, DEGs between CK-FS and 100MT-FS were annotated to the GO database, and they were primarily enriched in response to wounding (GO: 0009611), cell wall (GO: 0005618) and serine-type endopeptidase inhibitor activity (GO: 0004867) (Figure 4C). Interestingly, among the DEGs, most of the genes related to biological processes, cellular components and molecular functions were upregulated in the CK-HS vs. 100MT-HS and CK-FB vs. 100MT-FB comparisons, whereas the opposite trend was observed in CK-FS vs. 100MT-FS. Moreover, the molecular function category included the majority of the GO annotations, followed by biological process and cellular component.

**FIGURE 5:**
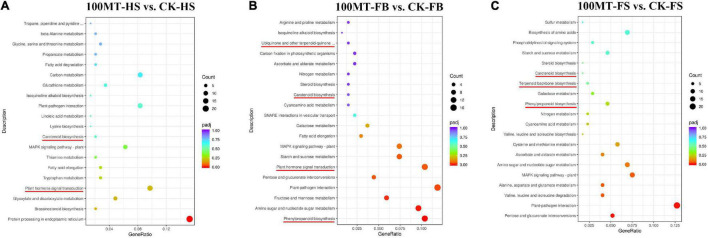
Representation of the top 20 statistically enriched KEGG terms among **(A)** CK-HS vs. 100MT-HS, **(B)** CK-FB vs. 100MT-FB and **(C)** CK-FS vs. 100MT-FS. KEGG category enrichment was computed using the R language, Cluster package, Biobase package, and *Q*-value package (*P* ≤ 0.01). Dot size indicates the number of DEGs in the corresponding pathway. The important pathways are indicated in red color.

Based on the pathways involved or the functions performed, DEGs were categorized using the KEGG database. The KEGG pathway database classifies biological metabolic pathways into three levels, with the first category including the second. The KEGG pathway enrichment analysis of differentially expressed genes revealed that there were three common pathways among the CK-HS vs. 100MT-HS, CK-FB vs. 100MT-FB and CK-FS vs. 100MT-FS comparisons. Among them, “MAPK signaling pathway” and “plant–pathogen interaction” were the highly significantly enriched pathways (Figures 5A–C). Moreover, three common pathways (“plant hormone signal transduction,” “fatty acid elongation,” and “isoquinoline alkaloid biosynthesis” pathways) were found between the CK-HS vs. 100MT-HS and CK-FB vs. 100MT-FB comparisons. Similarly, there were nine common pathways between the CK-FB vs. 100MT-FB and CK-FS vs. 100MT-FS comparisons. These nine common pathways included “phenylpropanoid biosynthesis,” “pentose and glucuronate interconversions,” “amino sugar and nucleotide sugar metabolism,” “starch and sucrose metabolism,” “galactose metabolism,” “cyanoamino acid metabolism,” “steroid biosynthesis,” “nitrogen metabolism,” and “ascorbate and aldarate metabolism”. The analysis of the top 20 significantly enriched KEGG pathways with DEGs is listed in Supplementary Table 4. Compared with the set of CK-FB vs. 100MT-FB and CK-FS vs. 100MT-FS, fourteen significantly enriched unique pathways were found in the set for CK-HS vs. 100MT-HS, which were “protein processing in endoplasmic reticulum,” “brassinosteroid biosynthesis,” “glyoxylate and dicarboxylate metabolism,” “tryptophan metabolism,” “thiamine metabolism,” “lysine biosynthesis,” “linoleic acid metabolism,” “glutathione metabolism,” “carbon metabolism,” “fatty acid degradation,” “propanoate metabolism,” “glycine, serine, and threonine metabolism,” “beta-alanine metabolism,” and “tropane, piperidine, and pyridine alkaloid biosynthesis” pathways. Similarly, compared with the set of CK-HS vs. 100MT-HS and CK-FS vs. 100MT-FS, seven significantly enriched pathways (“fructose and mannose metabolism,” “pentose and glucuronate interconversions,” “SNARE interactions in vesicular transport,” “carbon fixation in photosynthetic organisms,” “ubiquinone and other terpenoid-quinone biosynthesis,” “isoquinoline alkaloid biosynthesis,” and “arginine and proline metabolism”) were observed, which were unique to the set of CK-FB vs. 100MT-FB. Similarly, in comparison with the set of CK-HS vs. 100MT-HS and CK-FB vs. 100MT-FB, eight significantly enriched unique pathways were identified for CK-FS vs. 100MT-FS, which were “valine, leucine, and isoleucine degradation,” “alanine, aspartate, and glutamate metabolism,” “cysteine and methionine metabolism,” “valine, leucine, and isoleucine biosynthesis,” “terpenoid backbone biosynthesis,” “phosphatidylinositol signaling system,” “biosynthesis of amino acids,” and “sulfur metabolism” pathways. The presence of unique pathways at each developmental stage with respect to the control suggests their potential relative role in the floral aroma mechanism under the melatonin treatments. Furthermore, a total of 242, 196, and 283 DEGs were found by comparing the libraries CK-HS vs. 100MT-HS, CK-FB vs. 100MT-FB, and CK-FS vs. 100MT-FS, respectively.

### Transcription Factors Involved in Floral Scent Regulation

Several previous studies revealed that transcription factors (TFs) can regulate the mechanism of floral scent biosynthesis. Numerous TF families were identified among the significant DEGs using the plant TFDB database (Table 2). Under the melatonin treatment, 80, 106 and 72 differentially expressed transcription factors (TFs) were identified in CK-HS vs. 100MT-HS, CK-FB vs. 100MT-FB, and CK-FS vs. 100MT-FS, respectively. The transcription factors with the most copies were AP2 (66), WRKY (48), MYB (46), bHLH (34), bZIP (19), HSF (19), and GRAS (10). The melatonin treatment increased the expression of the majority of these transcription factor genes. Between CK-HS vs. 100MT-HS and CK-FB vs. 100MT-FB, the expression of the majority of transcription factors was upregulated and downregulated in CK-FS vs. 100MT-FS. Other TFs, such as ARF, Dof, GATA, TCP, and LSD, were also observed among these three stages of flower development under the melatonin treatment.

**TABLE 2:** Transcription factors identified in the significant DEGs.

	100MT-HS vs. CK-HS		100MT-FB vs. CK-FB		100MT-FS vs. CK-FS	
Transcription factors	Up	Down	Up	Down	Up	Down
AP2	17	1	33	0	2	13
ARF	1	0	0	0	1	1
bHLH	10	2	11	3	2	6
bZIP	3	0	5	1	0	10
Dof	1	0	1	1	1	2
GATA	0	0	4	0	0	1
GRAS	4	0	6	0	0	0
HSF	13	0	0	0	0	6
LSD	0	0	1	0	0	0
MYB	12	0	18	2	4	10
TCP	1	0	0	0	0	0
WRKY	15	0	20	0	1	12

### Key Scent-Related Genes Involved in Floral Aroma Synthesis Under the Melatonin Treatment

**FIGURE 6:**
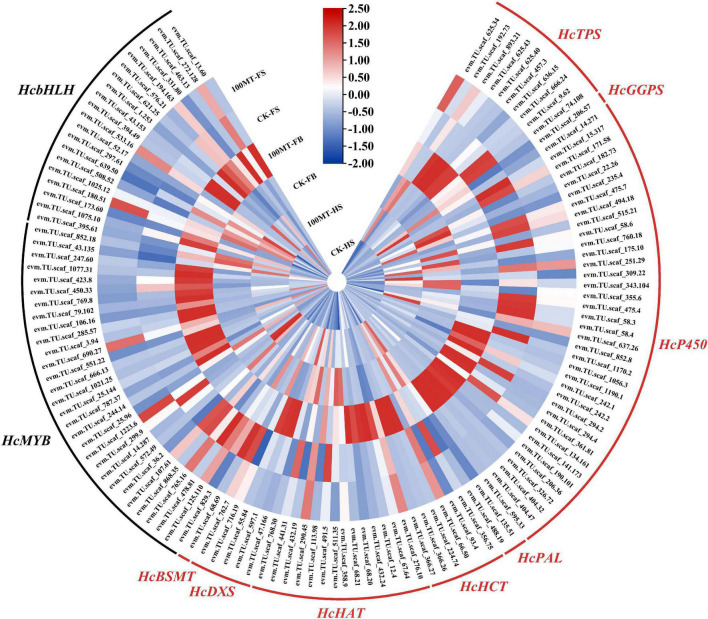
Heatmap of the expression levels of scent-related biosynthesis genes and MYB transcription factors identified in DEGs. Blue indicates low gene expression, white indicates no expression pattern, and red indicates high gene expression.

To reveal the regulatory mechanism of exogenous melatonin on scent metabolism-related genes in *H*. *coronarium* flowers, a cluster analysis of differentially expressed genes was employed to compare the expression levels of genes linked with floral scent production in three stages. A total of 126 differentially expressed genes were related to the floral scent mechanism, and their expression levels were revealed via a heatmap (Figure 6). Between CK-HS and 100-HS, 42 genes involved in the floral mechanism were significantly upregulated. A total of 65 genes were significantly upregulated in CK-FB vs. 100-FB. Interestingly, eight genes, including 6 transcription factors, were significantly upregulated in the fade stage compared to the corresponding control. Furthermore, 22, 29, and 6 transcription factors (MYB and bHLH) were upregulated in CK-HS vs. 100-HS, CK-FB vs. 100-FB, and CK-FS vs. 100-FS, respectively. The *HcTPS*, *HcBSMT*, *HcP450*, *HcHAT*, *HcGGPS*, *HcDXS HcPAL*, and *HcHCT* genes were mainly included among the upregulated genes.

### Network Analysis of Floral Volatile Compounds and Differentially Expressed Genes in Response to Melatonin Treatment

**FIGURE 7:**
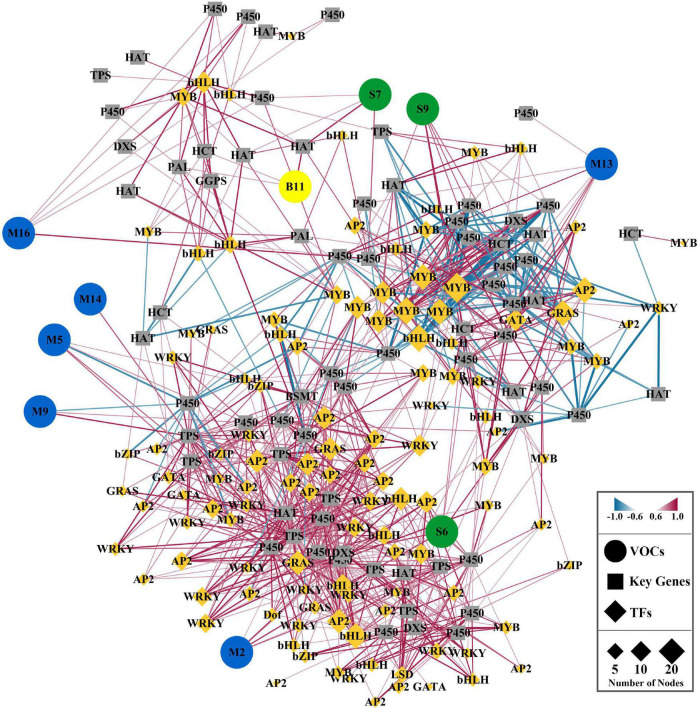
Correlation network analysis of significant DEGs with floral volatile compounds emitted in significantly higher amounts from *H*. *coronarium* flowers after melatonin treatment compared with the control flowers. Green, blue, and yellow color indicates the sesquiterpenes, monoterpenes and benzenoids. Volatile organic compounds are shown in circle and key genes in square, while transcription factors are indicated in diamond suit.

We performed a correlation test between the core volatile compounds and significant DEGs (floral scent-related genes and transcription factors) identified between the control flowers and melatonin-treated flowers to better understand the regulatory networks of floral volatile compounds (Figure 7 and Supplementary Table 5). The role of key transcription factors in floral scent metabolism was revealed based on a Spearman correlation analysis and further analyzed via cystoscopy. A gene-to-metabolite correlation analysis revealed that the MYB/bHLH transcription factors were the main regulators of floral volatile compounds. MYB (HcMYB_1021.25) was found to be the most significant transcription factor, and it was connected with 18 edges. Similarly, bHLHs (HcbHLH_331.80) and MYBs (HcMYB_107.61) were connected with 16 nodes each. Five transcription factors, MYB (HcMYB_1223.6 and HcMYB_106.16), bHLH (HcbHLH_43.153), GRAS (HcGRAS_211.12), and AP2 (HcAP2_519.11), were connected with 15 nodes. HcAP2_441.22 and HcGRAS_988.7 were interlinked with 14 nodes. Nine transcription factors (HcMYB_125.110, HcMYB_478.81, HcMYB_299.9, HcbHLH_576.21, HcbHLH_1075.10, HcAP2_26.297, HcAP2_432.10, HcAP2_579.37, and HcGRAS_405.14) were edged with 13 nodes, and four (HcbHLH_395.61, HcWRKY_71.45, HcAP2_99.133, and HcGATA_3.189) were interconnected with 12 nodes. In addition, HcMYB_3.94 was linked with 11 edges, and fourteen other transcription factors were connected with 10 nodes, suggesting that these are potential candidates for enhancing floral aroma production in *H*. *coronarium* flowers under the melatonin treatment.

### Validation of Scent-Related Genes via Quantitative Real-Time PCR

**FIGURE 8:**
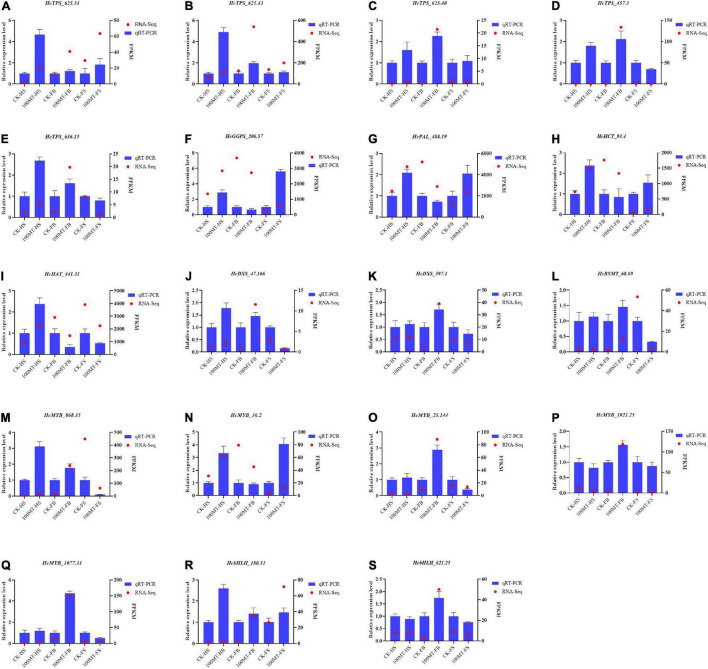
**(A–S)** Quantitative RT–PCR analysis of selected genes involved in scent-related pathways. GAPDH was used as an endogenous control. Data are presented as the mean ± SEM (*n* = 3). relative expression levels of target genes were calculated via the 2^–ΔΔCt^ method.

Quantitative RT–PCR was used to examine the expression profiles of selected genes involved in floral scent production (i.e., TPS, DXS, GGPS, HCT, HAT, BSMT, and PAL) or encoding MYB/bHLH TFs that were chosen from the RNA-seq data (Figure 8). The relative gene expression values determined by qRT–PCR were found to be strongly persistent with the FPKM determined by RNA-seq. *HcPAL* (*HcPAL_488.9*), gene showed a similar expression pattern with *HcHCT* (*HcHCT_93.4*) and *HcGGPS* (*HcGGPS_206.57*), with significantly higher expression during the HS and FS stages and lower expression during the FB stage compared with the control. Similarly, the transcript level of *HcDXS* (*HcDXS_47.166* and *HcDXS_597.1*) was similar to that of *HcBSMT* (*HcBSMT_68.69*). Overall, the majority of genes were upregulated during the half and full bloom stages of the flowers but downregulated during the fade stage of the flowers with respect to their control flowers. These results suggest that RNA-seq data are reliable and reproducible by independent methods, such as qRT–PCR.

## Discussion

Plant melatonin acts as an antioxidant and is involved in a variety of physiological processes, including seed germination, rooting, growth, osmoregulation, photosynthesis, protection against external stimuli, and gene expression regulation for a variety of physiological processes. *Hedychium coronarium* J. Koenig, commonly known as ginger lily, is an important ornamental/medicinal plant that is widely grown in tropical and semitropical regions of the world. Terpenoids (monoterpenes and sesquiterpenes) and benzenoids/phenylpropanoids are the main volatile components of *H*. *coronarium* flowers, and they are released in abundant amounts during the blooming period, which is consistent with previous reports (Yue et al., 2014, 2021; Ke et al., 2019; Zhou et al., 2021). However, the molecular regulatory mechanism of melatonin underlying floral aroma production remains unknown.

### Melatonin Enhanced Floral Aroma Production in *H*. *coronarium* Flowers

Several previous studies also showed that melatonin regulates several plant processes, including plant growth and development and senescence (Hu et al., 2020). Previously, RNA sequencing data revealed that the expression of numerous genes was influenced by melatonin regulation, causing changes in physiological activities. Exogenous melatonin application can boost endogenous melatonin contents and enhance its effects on a variety of physiological processes that lead to pleiotropic effects because of the signaling role of melatonin in plants (Byeon and Back, 2014; Fan et al., 2018; Xu et al., 2019). In the current study, the influence of melatonin on *H*. *coronarium* flowers was evaluated using the HS–SPME–GC–MS method, and its effect on individual compounds was evaluated. We observed that melatonin application substantially enhanced floral scent production in *H*. *coronarium* relative to that of the control flowers. Under the melatonin treatment, the amount of certain volatile compounds increased during the half bloom, full bloom and fade stages of the flowers compared to the control group. Similarly, in *Camellia sinensis*, exogenous melatonin and gibberellin application significantly affected terpenoid synthesis and hormone signal transduction pathways (Di et al., 2019). The effect of melatonin varied from species to species and based on the study objectives. In tomato, melatonin promotes anthocyanin accumulation and fruit ripening after harvesting (Hardeland, 2016; Sun et al., 2016). Similarly, melatonin induced significant changes in compounds during developmental processes, and it played a pleiotropic orchestrating role in lignin formation in tea plants (Li et al., 2020). In grape fruits, exogenous melatonin and ABA significantly increase flavonol accumulation by upregulating the expression of flavonoid biosynthesis genes (Yang et al., 2020). These results indicate that melatonin significantly triggered the mechanism of floral aroma production.

### Melatonin Regulates the Complex Regulatory Network of Floral Scent Production

Floral aroma production and emission is a complex and dynamic mechanism that involves the coordination of several genes that are regulated by various transcription factors (Pichersky and Gershenzon, 2002; Gershenzon and Dudareva, 2007; Nagegowda and Gupta, 2020). By analyzing the integrated metabolome and transcriptome data, we can better understand the mRNA abundance and gene profiles of *H*. *coronarium* flowers treated with melatonin. Our findings showed that the gene expression trends of qRT–PCR strongly supported the RNA-seq data. Under the melatonin treatment, several DEGs were significantly upregulated, suggesting that these genes can play crucial roles in the floral aroma mechanism. Among the differentially expressed genes, key scent-related enzyme genes, such as TPS, DXS, BSMT, HAT, GGPS, PAL, and HCT, were significantly upregulated. Previous studies showed that the enzyme genes TPS, DXS, BSMT, HAT, GGPS, and PAL are the key genes in the terpenoid and benzenoid/phenylpropanoid pathways and produce numerous important secondary metabolites (Pichersky and Gershenzon, 2002; Aharoni et al., 2003; Phillips et al., 2007; Abbas et al., 2019). Similarly, in *C. sinensis*, exogenous melatonin application substantially influenced the terpenoid synthesis pathway by triggering the expression level of related genes (Di et al., 2019). Furthermore, the gene-to-metabolite analysis showed that these key enzyme genes were linked to numerous nodes of certain volatile compounds, suggesting their potential role in specific volatile synthesis. The RNA-seq and qRT–PCR results showed that the expression of the aforementioned key enzymes was significantly influenced by the melatonin treatment (Figure 8). In Arabidopsis and strawberry, the overexpression of key enzyme genes in terpenoid pathways significantly enhanced the terpenoid volatile compounds, and downregulation resulted in the opposite trend (Aharoni et al., 2003, 2004; Tholl, 2006). In *Vitis vinifera* cv. Kyoho, melatonin and ABA application triggered flavonoid biosynthesis by influencing the expression level of structural flavonoid biosynthesis genes (Yang et al., 2020). Our findings are in line with previous reports that melatonin significantly influenced the expression level of certain genes involved in several biological processes (Arnao and Hernández-Ruiz, 2019; Cecon et al., 2019). Hence, we can hypothesize that melatonin is involved in the regulation of certain floral volatile compounds by influencing the expression of structural volatile synthesis genes, which can be validated via further in-depth molecular intervention.

### Melatonin Could Regulate MYB/bHLH Transcription Factors Involved in the Floral Aroma Mechanism

**FIGURE 9:**
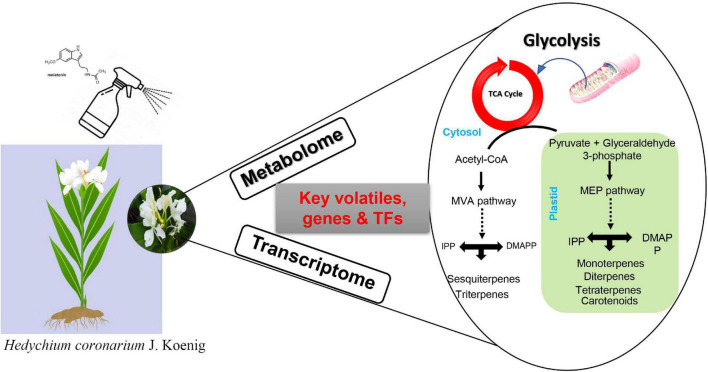
Summary model of the regulatory pathway of exogenous melatonin in floral scent formation in *H. coronarium*.

Floral scent is produced in various parts of the plant and abundantly released, particularly from flowering parts. Several previous studies revealed that transcription factors play crucial roles in floral aroma biosynthesis (Zvi et al., 2012; Liu et al., 2017; Sasaki, 2018). Evidence has shown that the MYB/bHLH transcription factor regulates floral aroma metabolism by binding to the promoter region of enzyme genes (terpene synthases) (Deluc et al., 2006; Bedon et al., 2010; Colquhoun et al., 2010; Amarr et al., 2017). Among the significant differentially expressed genes, 12, 18, and 4 HcMYB transcription factor genes were significantly upregulated in the CK-HS vs. 100MT-HS, CK-FB vs. 100MT-FB, and CK-FS vs. 100MT-FS comparisons, respectively, whereas 2 and 10 HcMYB transcription factors were downregulated in the CK-FB vs. 100MT-FB and CK-FS comparisons, respectively. Furthermore, the Spearman correlation analysis showed that HcMYB/bHLH were among the main transcription factors interlinked with several nodes of major floral volatile components, indicating that these genes are candidate genes that activate floral aroma biosynthesis under the melatonin treatment. Similar findings were obtained in *C. sinensis*, where MYB transcription factors regulate lignin biosynthesis under the melatonin treatment (Han et al., 2021). Interestingly, in gene-to-metabolite correlation analysis, we observed that HcMYB_1021.25 (HcMYB248) was among the key HcMYB transcription factors connected with 18 nodes. Our previous study regarding the genome-wide analysis of HcMYB revealed that *HcMYB248* was the key transcription factor controlling floral aroma biosynthesis by binding to the promoter region of structural synthesis genes, and its suppression resulted in reduced floral volatile contents by downregulating the expression of structural volatile synthesis genes (Abbas et al., 2021a). In petunia, ODO1, EOBI, and EOBII regulate the benzenoid biosynthetic pathways by regulating related gene expression (Verdonk et al., 2005; Van Moerkercke et al., 2011; Spitzer-Rimon et al., 2012). In *Senecio cruentus*, several R2R3-MYB transcription factors are involved in the regulation of anthocyanin contents (Cui et al., 2021). Our findings are consistent with previous findings that exogenous melatonin application regulates secondary metabolism (Fan et al., 2018). The summary of current study is summarized in Figure 9. Our findings showed that MYB transcription factors are the key regulators of floral aroma metabolism in *H*. *coronarium*.

## Conclusion

In the current study, we observed that exogenous melatonin application significantly enhanced the floral volatile contents in *H*. *coronarium* flowers. We used metabolomics and transcriptomics approaches to analyze the phytochemical and transcriptional changes that occur during the flower developmental process of *H*. *coronarium* flowers after melatonin treatments. The data showed that the production of certain volatile organic compounds was significantly increased, and hundreds of differentially expressed genes were identified and further analyzed. A total of 76 DEGs were significantly upregulated and involved in the complex floral aroma biosynthesis mechanism. The expression of key genes associated with the floral volatile mechanism was verified by RT–qPCR. A gene-to-metabolite correlation analysis using Cytoscape was performed to identify the key regulators of floral volatile compounds, which showed that the transcription factors HcMYB/bHLH were mainly interlinked with several nodes and considered potential candidates for the regulation of floral volatile compounds in *H*. *coronarium* flowers. In short, although the current study has some limitations, our results provide valuable insights into the regulatory mechanism of melatonin in floral aroma biosynthesis in *H*. *coronarium* flowers.

## Data Availability Statement

The datasets presented in this study can be found in online repositories. The names of the repository and accession number can be found below: https://www.ncbi.nlm.nih.gov/sra/PRJNA777930.

## Author Contributions

FA, YK, YF, and RY conceived and designed the concept, and revised and finalized the manuscript. FA, YK, and JH performed the experiments and did the formal analysis. FA and YZ analyzed the data and drafted the manuscript. All authors endorsed the final version of the manuscript.

## Conflict of Interest

The authors declare that the research was conducted in the absence of any commercial or financial relationships that could be construed as a potential conflict of interest.

## Publisher’s Note

All claims expressed in this article are solely those of the authors and do not necessarily represent those of their affiliated organizations, or those of the publisher, the editors and the reviewers. Any product that may be evaluated in this article, or claim that may be made by its manufacturer, is not guaranteed or endorsed by the publisher.

## Footnotes

1
http://www.repeatmasker.org/cgi-bin/RepeatProteinMaskRequest

## Supplementary Material

Supplementary Figure 1 Correlation analysis of floral volatile compounds between the treated and untreated flowers.

Supplementary Figure 2 Volcano plot of the DEGs identified in CK-HS vs. 100MT-HS, CK-FB vs. 100MT-FB, and CK-FS-100MT-FS.

Supplementary Figure 3 Transpose super-families significantly influenced under melatonin treatments.

Supplementary Table 1 Analysis of the sequencing data statistics.

Supplementary Table 2 Statistics for the sample and reference genome comparisons.

Supplementary Table 3 List of the top biological processes observed in the GO classification.

Supplementary Table 4 List of significantly enriched pathways of DEGs among CK-HS vs. 100MT-HS, CK-FB vs. 100MT-FB and CK-FS vs. 100MT-FS.

Supplementary Table 5 List of primers used in the assay.
