# Supplementary figures and images for: Metabolite and Transcriptome Profiling Analysis Revealed That Melatonin Positively Regulates Floral Scent Production in Hedychium coronarium
Source: Front Plant Sci. 2021 Dec 17;12:808899. doi: 10.3389/fpls.2021.808899 (PMC8719004; doi:10.3389/fpls.2021.808899)

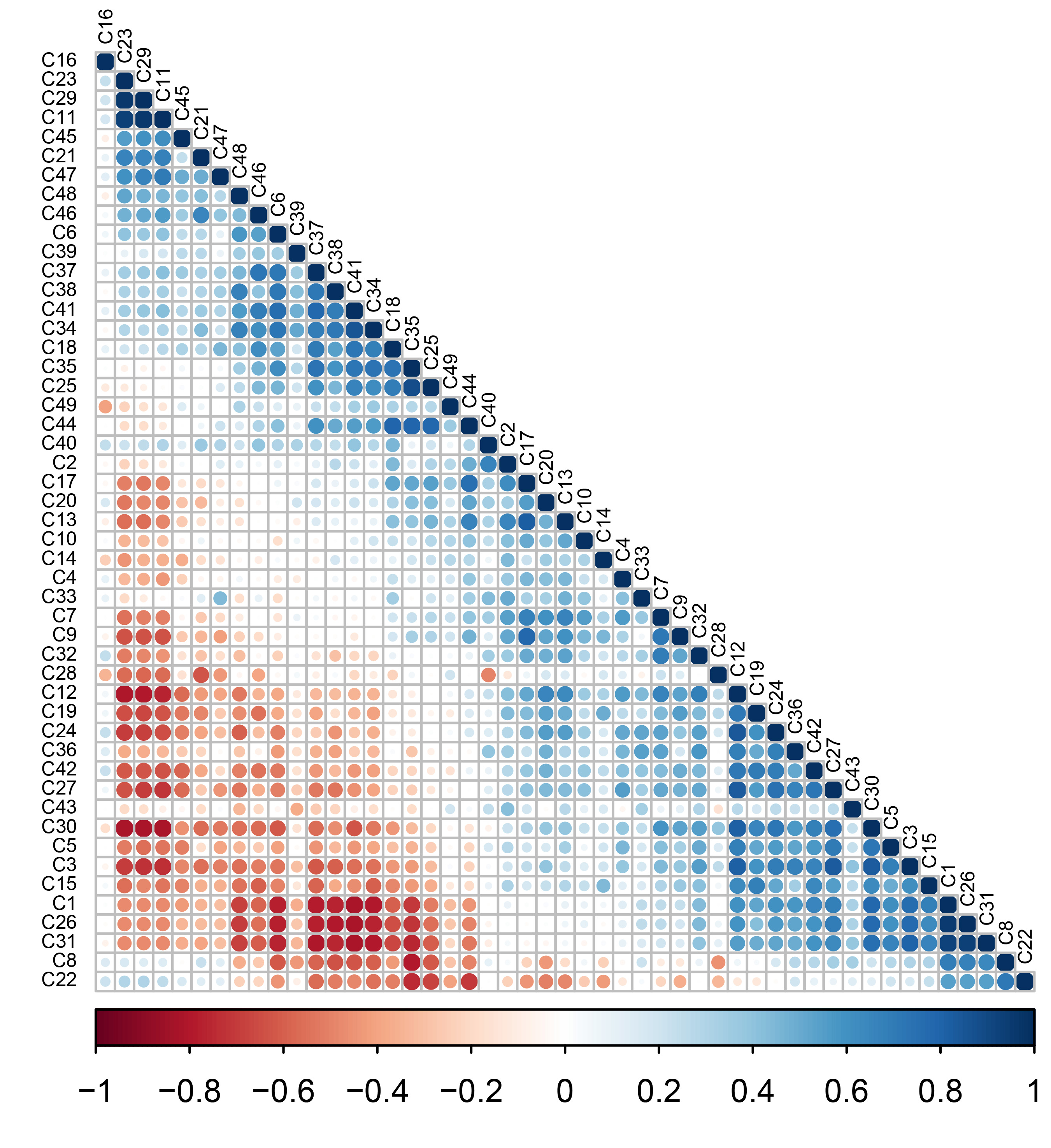

Supplement: Supplementary Figure 1 — Correlation analysis of floral volatile compounds between the treated and untreated flowers. [file Image_1.JPEG]

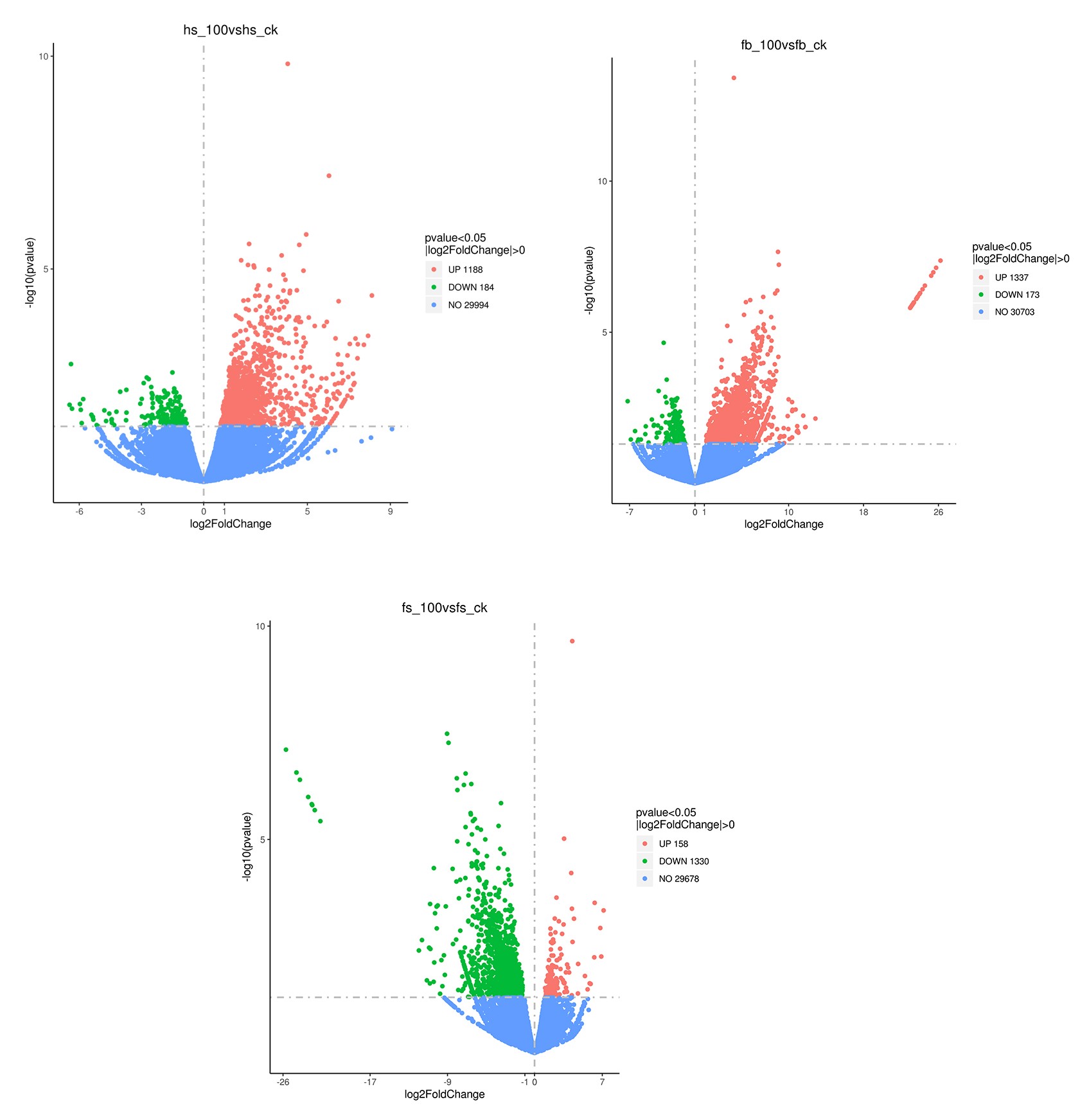

Supplement: Supplementary Figure 2 — Volcano plot of the DEGs identified in CK-HS vs. 100MT-HS, CK-FB vs. 100MT-FB, and CK-FS-100MT-FS. [file Image_2.JPEG]

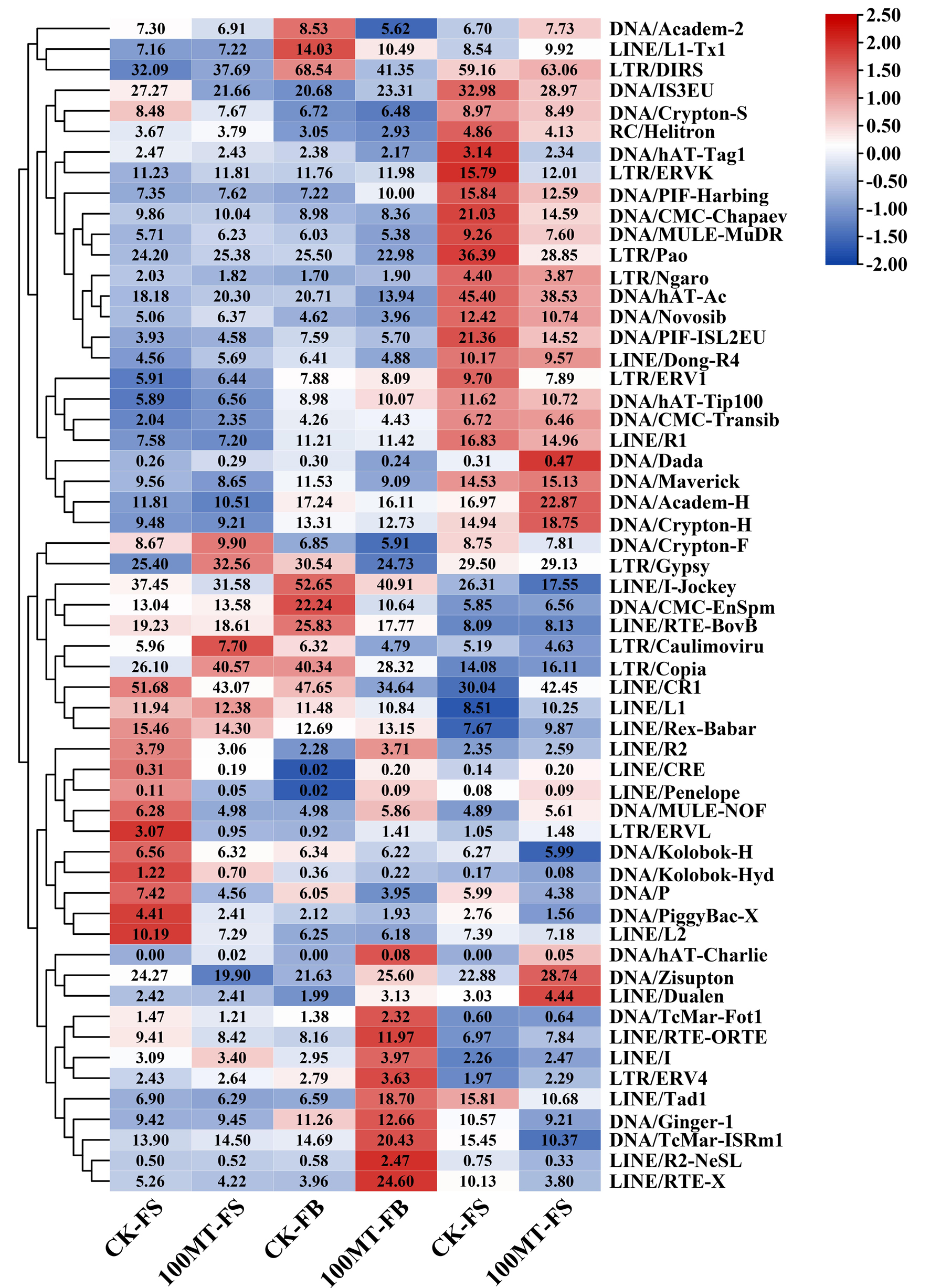

Supplement: Supplementary Figure 3 — Transpose super-families significantly influenced under melatonin treatments. [file Image_3.JPEG]
